# Marrow adipose tissue is increased in overweight and obese women with PCOS independently of hyperandrogenism related obesity and metabolic disorders

**DOI:** 10.3389/fendo.2023.1168806

**Published:** 2023-06-09

**Authors:** Li Xu, Min Min, Xintong Li, Glen M. Blake, Kaiping Zhao, Xiangyan Ruan, Xiaoguang Cheng

**Affiliations:** ^1^ Department of Radiology, Beijing Jishuitan Hospital, Beijing, China; ^2^ Department of Gynecology, Aviation General Hospital, Beijing, China; ^3^ Department of Gynecological Endocrinology, Beijing Obstetrics and Gynecology Hospital, Capital Medical University, Beijing Maternal and Child Health Care Hospital, Beijing, China; ^4^ Biomedical Engineering Department, King’s College London, London, United Kingdom; ^5^ Department of Medical Record Management and Statistics, Beijing Jishuitan Hospital, Beijing, China

**Keywords:** bone marrow adipose tissue, polycystic ovary syndrome, hyperandrogenism, obesity, metabolic syndrome

## Abstract

**Purpose:**

This study aimed to investigate the increase in bone marrow adipose tissue (BMAT) in overweight and obese women with polycystic ovary syndrome (PCOS) and its relationship with hyperandrogenism, obesity, and metabolic disorders.

**Methods:**

The study included 87 overweight or obese women with PCOS (mean age 29 ± 4 years), as well as 87 age-matched controls recruited from a separate population study. All PCOS patients were measured for anthropometric features, abdominal adipose tissue areas, BMAT, biochemistry, and sex hormones. BMAT was compared between the PCOS patients and controls. In PCOS patients, subgroup comparisons of BMAT and its associations with body adiposity indices, biochemistry, and sex hormones were analyzed. The odds ratios (ORs) of elevated BMAT (defined as BMAT ≥ 38%) were calculated.

**Results:**

On average BMAT was increased by 5.6% ( ± 11.3%) in PCOS patients compared to controls. BMAT were significantly higher in the upper tertiles of total cholesterol (TC) and low density lipoprotein-cholesterol (LDL-C). BMAT was not correlated with abdominal adiposity indices or biochemistry except for LDL-C (r = 0.253—0.263, *p* = 0.014—0.018). LDL-C was not significantly different between the normal and abnormal androgen PCOS subgroups (*p* = 0.10-0.887). LDL-C, follicle stimulating hormone (FSH), and total testosterone (TT) were risk factors for elevated BMAT, with ORs of 1.899 (*p* = 0.038-0.040), 1.369 (*p* = 0.030-0.042), and 1.002 (*p* = 0.040-0.044) for each unit increase, respectively.

**Conclusion:**

BMAT was increased in overweight and obese PCOS patients, but the increase in BMAT was not associated with the hyperandrogenism related obesity or metabolic disorders.

## Introduction

Polycystic ovary syndrome (PCOS) is the most common endocrine disorder among women of reproductive age. Hyperandrogenism is the most consistent endocrine feature in PCOS and is likely to play a key role in the etiology of the condition (1, 2). PCOS is known to be associated with metabolic abnormalities such as insulin resistance, dyslipidemia, chronic low-grade inflammation, and arterial hypertension (3, 4). Epidemiological data revealed that 30-70% of women with PCOS are either overweight or obese (5–7). Obese women with PCOS have a more severe phenotype than those who are less obese, with more severe menstrual irregularities, infertility, miscarriage, and clinical hyperandrogenism, glucose intolerance and/or type 2 diabetes mellitus, and metabolic syndrome (8, 9).

In PCOS, the effects of hyperandrogenism on the adipose tissue have been exhibited within the white adipose tissue and brown adipose tissue (10). Bone marrow adipose tissue (BMAT) is a type of unique adipose tissue in humans, and increases with age and becomes a prominent component of bone marrow. It is well known that increased BMAT is associated with decreased bone mass and osteoporosis (11, 12). The accumulation of BMAT can be accelerated by different pathophysiological conditions, including obesity, diabetes, glucocorticoid therapy, anorexia nervosa, and Cushing’s syndrome (13). However, it is not known whether PCOS-related obesity and metabolic abnormalities in women lead to an increase in BMAT. The adverse effect of hyperandrogenism on increased BMAT, confirmed in rodent models, has not been verified in women with PCOS (14, 15). In this study, we aimed to explore the phenotype of BMAT in overweight or obese young PCOS patients and furthermore to investigate the relationships between the increase in BMAT and hyperandrogenism, obesity, and metabolic disorders in PCOS.

## Methodology

### Study subjects

This was a prospective, single centered, clinical study. All participants with overweight or obese PCOS were recruited from consecutive patients visiting the gynecological endocrinology clinic of the Beijing Obstetrics and Gynecology Hospital between February and December 2019. According to the Chinese adult overweight and obesity prevention and control guidelines (16), overweight or obese was defined as a BMI ≥ 24 kg/m^2^ (overweight: 24 kg/m^2^ ≤ BMI < 28 kg/m^2^; obese: BMI ≥ 28 kg/m^2^). PCOS was diagnosed using the Rotterdam ESHRE/ASRM revised 2003 consensus criteria (17) as follows: oligo-ovulation, anovulation, or irregular uterine bleeding; having one of the two following abnormalities: ① clinical manifestations of hyperandrogenism or hyperandrogenemia, or ② typical polycystic ovaries shown on ultrasound (the number of follicles with a diameter of 2-9mm ≥ 12 in unilateral or bilateral ovaries, and/or ovarian volume ≥ 10 cm^3^). All the other diseases that may cause hyperandrogenism or abnormal ovulation were excluded, including Cushing’s syndrome, non-classical congenital adrenal hyperplasia (NCCAH), ovary or adrenal gland tumors that secrete androgens, functional hypothalamic amenorrhea, hyperprolactinemia, and premature ovarian insufficiency (POI). The inclusion criteria were: aged from 16 to 40 years; overweight or obese PCOS patient; waist circumference (WC) ≥ 80cm. A total of 87 eligible women were finally recruited for participation in the study.

Eighty-seven age-matched controls were recruited from a separate population study to investigate the degeneration of spine and knee (China Action on Spine and Hip study, CASH). All participants were healthy adults who had lived in Beijing for more than 5 years, and the inclusion criteria for that study were previously published (18). Participants who had oligo-ovulation, anovulation, or irregular uterine bleeding were excluded. Random case-control matching was used to perform the age-matched control sampling; 77 samples attained exact matching and 10 samples achieved fuzzy matching (± 1year).

The regional ethics committee of the Beijing Obstetrics and Gynecology Hospital, Capital Medical University approved this study (Protocol number 2018-ky-011-01; Registration number ChiCTR1900020986). All participants provided signed, informed consent. The study was carried out in accordance with The Code of Ethics of the World Medical Association (Declaration of Helsinki).

All the overweight and obese women with PCOS received blood serum tests, and on the same day, both routine and chemical shift encoded magnetic resonance imaging (CSE-MRI) scans were performed for the measurements of abdominal adipose tissue and BMAT. All controls underwent a quantitative computed tomography (QCT) scan for the measurements of abdominal adipose tissue, and 40 controls underwent a CSE-MRI scan for the measurement of BMAT on the same day.

### MR scan and measurements

MR scans were obtained on a clinical 3T whole-body MRI system (Ingenia 3.0T; Philips Healthcare, Best, The Netherlands) with a 32-channel dS Torso Body coil. The routine abdominal T2-weighted turbo spin echo (TSE) axil sequence was acquired with the parameters as: time of repetition (TR) = 3500 ms, time to echo (TE) = 110 ms, slice thickness = 6 mm, slice gap = 2 mm, field of view (FOV) = 440 × 385 mm2, voxel size = 1.6 × 1.6 mm2, number of signals acquired (NSA) = 1. T2-weighted TSE axial images were analyzed by using the OsiriX MD version 6 software (Pixmeo SARL, Bernex, Switzerland). Total adipose tissue (TAT), visceral adipose tissue (VAT), and subcutaneous adipose tissue (SAT) areas (cm2) were semi-automatically measured at the level of the umbilicus.

CSE-MRI was obtained by mDixon-quant, a 3D spoiled gradient-echo sequence using multiple acquired echoes to generate water, fat, T2*, R2*, and in-phase and oppose-phase images synthesized from the water-fat images. The scan parameters of the single breath-hold mDixon-quant were described previously (19). The CSE-MRI dataset were processed with ISP version 7 software (Philips Healthcare, Best, the Netherlands). Region of interests was drawn manually on the central L2, L3, and L4 axial proton density fat fraction (PDFF) map images, and the mean PDFF (%) of each vertebra was evaluated. BMAT was calculated as the mean value of L2 to L4 PDFF.

### QCT scan and measurements

According to the protocols of the CASH study, abdominal QCT scan was performed on a Toshiba CT scanner (Aquilion Prime ESX-302A; Toshiba Medical Systems Corporation, Otawara, Japan) with a five-rod calibration phantom (Model 3 phantom; Mindways Inc., Austin, TX, USA) placed beneath the subject and scanned simultaneously (18).

The CT volumetric datasets were transferred to a QCT workstation and analyzed using Mindways QCT PRO tissue composition module software version 4.2. As described previously (20), TAT, VAT, and SAT areas (cm2) were semi-automatically measured at the level of the umbilicus.

### Blood serum analyses

A fasting, morning blood sample was taken from each overweight or obese PCOS patient on the 2nd - 5th day after spontaneous menses or on any day for those with amenorrhea for more than 3 months. The blood samples were assayed for triglyceride (TG, mmol/L), total cholesterol (TC, mmol/L), high density lipoprotein-cholesterol (HDL-C, mmol/L), low density lipoprotein-cholesterol (LDL-C, mmol/L), and glucose (mmol/L) by using a Synchron LX-20 automated analyzer (Bechman Coulter, CA, US). Sex hormones and insulin (pmol/L) assays were conducted by chemiluminescence immunoassay using an ADVIA Centaur XP automated analyzer (Siemens Healthcare Diagnostics, CA, US). Sex hormones that were measured included follicle stimulating hormone (FSH, IU/L), luteinizing hormone (LH, IU/L), estradiol (pg/mL), progesterone (ng/mL), total testosterone (TT, pg/mL), free testosterone (FT, pg/mL), sex hormone-binding globulin (SHBG, nmol/L), and prolactin (ng/mL). The ratio of LH to FSH (LH/FSH ratio = LH (IU/L)/FSH (IU/L)) was then calculated.

### Statistical analysis

Distributions of the variables were examined for departure from normality by the Shapiro-Wilk test. Data were presented as the mean ± standard deviation (SD), median (interquartile, IQR), and 95% confidence interval (CI). For the inter-group comparisons between the PCOS patients and controls, the Student t test was used for normally distributed variables and the Mann-Whitney U test was used for skewed variables.

A one-way ANOVA or Mann-Whitney U test was used to compare the differences of adiposity indices and biochemistry measurements among the PCOS subgroups stratified by the level of androgens, LH, and LH/FSH ratio, respectively. Pearson and partial correlations were calculated between BMAT and body adiposity indices and biochemistry measurements. BMAT was also compared between the tertiles of body adiposity indices and biochemistry measurements.

Multivariate linear regression analysis was performed to estimate the contribution of each sex hormone to BMAT, without (model 1) and with adjustments for age, BMI (model 2), and additionally for biochemistry (model 3). BMAT was considered to be elevated when BMAT was higher than the 90% upper limit of the BMAT range of the controls. The odds ratios (ORs) of elevated BMAT was calculated by univariate logistic regression models without (model 1) and with adjustments for age, BMI, and biochemistry (model 2). Furthermore, cut off values for diagnosing elevated BMAT were calculated for those significant risk factors by the maximum value of Youden’s Index (Youden’s Index = sensitivity + specificity - 1) using the receiver operating characteristic (ROC) curve, and those risk factors were then transformed into dichotomous variables according to the cut off values. Multivariate binary logistic regression analysis was performed to evaluate the predictors for elevated BMAT.

All data managements and analyses were completed using SPSS version 22.0 (SPSS Inc., Chicago, IL, USA). A two-sided p value of < 0.05 was considered to indicate statistical significance.

## Results

### Discrepancy of adiposity indices between overweight and obese PCOS patients and age-matched controls

The characteristics and adiposity indices of the overweight and obese PCOS patients and controls are presented in **Table 1**. Compared to the age-matched controls, a larger SAT and TAT area was found along with the higher weight and BMI in the overweight or obese PCOS patients. Although VAT area was also found to be larger, the difference was insignificant. BMAT was higher in the overweight or obese PCOS patients with a mean difference of 5.6% ( ± 11.3%).

**Table 1:** Comparisons of characteristics and adiposity indices between overweight and obese PCOS patients and age-matched controls.

		Mean ± SD	Median (P_25_, P_75_)	95%CI	Normality test (Shapiro-Wilk)	Inter-group comparisons (Student t test/Mann-Whitney U test)
Age (years)	Group _POCS_	29 ± 4	29 (27, 31)	20—38	*p* = 0.056	*p* = 0.208
	Group _control_	29 ± 4	29 (27, 32)	21—38	*p* = 0.174	
	Difference _PCOS-control_	0 ± 0	0 (0, 0)	-1—1	*p* < 0.001	
Height (m)	Group _POCS_	1.62 ± 0.05	1.62 (1.60, 1.66)	1.52—1.72	*p* = 0.283	*p* = 0.141
	Group _control_	1.61 ± 0.05	1.62 (1.58, 1.64)	1.52—1.71	*p* = 0.195	
	Difference _PCOS-control_	0.01 ± 0.06	0.02 (-0.03—0.05)	-0.12—0.11	*p* = 0.057	
Weight (kg)	Group _POCS_	76.2 ± 10.1	75.0 (70.0, 80.0)	63.0—105.4	*p* < 0.001	***p* < 0.001**
	Group _control_	59.4 ± 9.2	58.0 (53.0, 65.0)	45.0—84.0	*p* = 0.003	
	Difference _PCOS-control_	16.8 ± 12.8	16.0 (9.0, 24.0)	-10.4—43.6	*p* = 0.20	
BMI (kg/m^2^)	Group _POCS_	28.9 ± 3.5	28.3 (26.2, 30.9)	24.1—37.0	*p* < 0.001	***p* < 0.001**
	Group _control_	22.8 ± 3.4	22.5 (20.1, 24.7)	18.0—30.3	*p* = 0.003	
	Difference _PCOS-control_	6.1 ± 4.4	5.3 (3.4, 9.3)	-3.3—15.7	*p* = 0.20	
VAT area (cm^2^)	Group _POCS_	77.8 ± 27.4	73.5 (57.4, 97.3)	34.7—130.6	*p* = 0.004	*p* = 0.233
	Group _control_	73.8 ± 26.0	70.1 (57.0, 84.3)	34.5—160.0	*p* < 0.001	
	Difference _PCOS-control_	4.0 ± 33.4	2.6 (-15.1, 28.6)	-69.9—63.7	*p* = 0.20	
SAT area (cm^2^)	Group _POCS_	294.2 ± 84.1	274.8 (228.9, 344.7)	171.9—540.5	*p* < 0.001	***p* < 0.001**
	Group _control_	167.5 ± 67.8	162.3 (126.3, 213.3)	40.7—327.2	*p* = 0.002	
	Difference _PCOS-control_	126.7 ± 104.3	120.0 (61.4, 203.5)	-84.2—359.4	*p* = 0.20	
TAT area (cm^2^)	Group _POCS_	372.0 ± 96.5	357.8 (296.5, 423.8)	218.1—675.0	*p* < 0.001	***p* < 0.001**
	Group_control_	241.3 ± 87.2	233.6 (179.9, 282.0)	82.1—456.1	*p* < 0.001	
	Difference _PCOS-control_	130.7 ± 121.0	121.7 (50.5, 211.7)	-118.2—393.9	*p* = 0.20	
BMAT (%)	Group _POCS_	34.5 ± 8.2	35.6 (29.0, 39.5)	18.5—51.0	*p* = 0.868	***p* = 0.003**
	Group_control_^a^	29.0 ± 7.0	28.1 (26.4, 33.3)	14.2—46.3	*p* = 0.327	
	Difference _PCOS-control_^a^	5.6 ± 11.3	4.8 (-2.5, 10.4)	-14.8—33.9	*p* = 0.20	

^a^BMAT was measured in a total of 40 subjects in the control group, and therefore the comparison of BMAT between PCOS patients and controls was obtained in 40 pairs.

SD, standard deviation; CI, confidence interval; BMI, body mass index; VAT, visceral adipose tissue; SAT, subcutaneous adipose tissue; TAT, total adipose tissue; BMAT, bone marrow adipose tissue.

The bold values mean statistically significant (p < 0.05).

### Changes of sex hormones in overweight and obese PCOS patients and their relations to adiposity indices and biochemistry

The values of biochemistry and sex hormone measurements of the overweight or obese PCOS patients are presented in **Table 2**. About one-third to half of the patients had abnormal triglyceride and cholesterol levels. Two-thirds of the patients had a decreased SHBG, and more than 20% of the patients had increased FT (56.3%), TT (34.5%), LH (24.1%), and LH/FSH ratio (26.5%).

**Table 2:** Levels of biochemistry measurements and sex hormones in overweight and obese PCOS patients.

	Mean ± SD	Median (P_25_, P_75_)	95% CI	Normality test (Shapiro-Wilk)	Reference Standard	Number of subjects (percentage) within normal range
TG (mmol/L)	2.06 ± 1.34	1.72 (1.27, 2.49)	0.59—6.97	*p* < 0.001	≤ 1.70	N = 42 (48.3%)
TC (mmol/L)	5.01 ± 0.97	4.82 (4.2, 5.66)	3.48—7.14	*p* = 0.079	≤ 5.18	N = 52 (59.8%)
HDL-C (mmol/L)	1.09 ± 0.21	1.05 (0.96, 1.25)	0.72—1.64	*p* = 0.023	> 1.04	N = 45 (51.7%)
LDL-C (mmol/L)	3.08 ± 0.80	3.0 (2.45, 3.68)	1.72—4.93	*p* = 0.20	≤ 3.34	N = 57 (65.5%)
Glucose (mmol/L)	5.17 ± 0.58	5.07 (4.81, 5.44)	4.25—6.7	*p* = 0.018	≤ 6.10	N = 83 (92.2%)
Insulin (pmol/L)	109.91 ± 70.83	89.59 (64.23, 149.43)	13.96—335.17	*p* = 0.001	21—174	N =70 (80.5%)
FSH (IU/L)	5.98 ± 1.76	5.97 (4.91, 7.25)	2.27—9.37	*p* = 0.942	2.5—10.2	N = 85 (97.7%)
LH (IU/L)	9.38 ± 6.0	7.71 (4.95, 11.99)	1.91—25.33	*p* < 0.001	1.9—12.5	N = 66 (75.9%)
LH/FSH ratio	1.59 ± 0.91	1.28 (0.98, 2.05)	0.40—4.30	*p* < 0.001	≤ 2	N = 64 (73.5%)
Estradiol (pg/mL)	60.43 ± 45.01	49.91 (42.81, 61.3)	18.22—209.73	*p* < 0.001	19.5—144.2	N = 81 (93.1%)
Progesterone (ng/mL)	1.08 ± 3.17	0.49 (0.27, 0.75)	0.03—7.59	*p* < 0.001	≤ 1.4	N = 80 (92.0%)
TT (pg/mL)	486.26 ± 216.78	439.31 (314.4, 604.54)	208.52—1065.7	*p* < 0.001	90—550	N = 57 (65.5%)
FT (pg/mL)	9.02 ± 4.87	8.98 (5.93, 12.4)	1.08—20.58	*p* = 0.101	0.8—7.4	N = 38 (43.7%)
SHBG (nmol/L)	48.9 ± 58.06	26.6 (16.8, 45.7)	9.02—250.0	*p* < 0.001	> 34.3	N = 29 (33.3%)
Prolactin (ng/mL)	11.42 ± 5.49	10.44 (8.13, 13.35)	4.43—31.07	*p* < 0.001	2.8—29.2	N = 85 (97.7%)

SD, standard deviation; CI, confidence interval; TG, triglyceride; TC, total cholesterol; HDL-C, high density lipoprotein-cholesterol; LDL-C, low density lipoprotein-cholesterol; FSH, follicle stimulating hormone; LH, luteinizing hormone; LH/FSH, luteinizing hormone/follicle stimulating hormone; TT, total testosterone; FT, free testosterone; SHBG, sex hormone-binding globulin.

**Figure 1:**
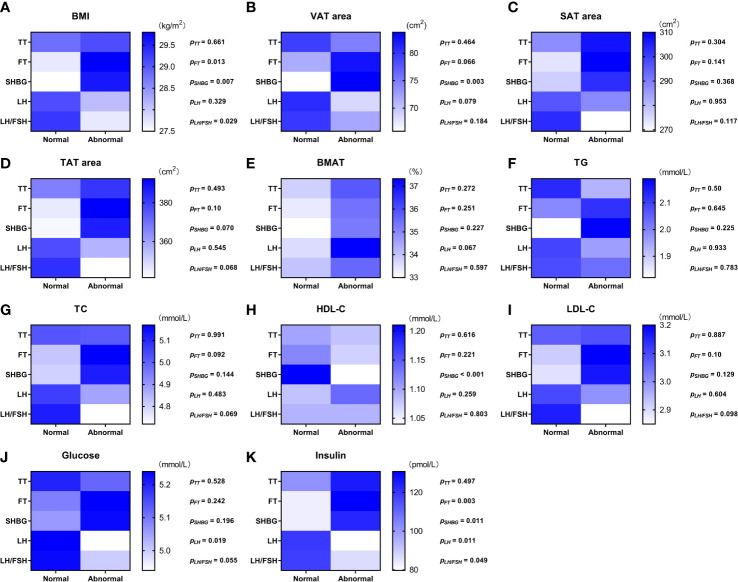
Color scale diagram of the values of adiposity indices and biochemistry measurements in each overweight and obese PCOS patient subgroup divided according to the value of sex hormones. The overweight and obese PCOS patients were divided into two subgroups according to the value (normal/abnormal) of total testosterone (TT), free testosterone (FT), sex hormone-binding globulin (SHBG), luteinizing hormone (LH), and ratio of luteinizing hormone to follicle stimulating hormone (LH/FSH ratio), respectively. The values of body mass index (BMI) **(A)**, visceral adipose tissue (VAT) area **(B)**, abdominal subcutaneous adipose tissue (SAT) area **(C)**, abdominal total adipose tissue (TAT) area **(D)**, bone marrow adipose tissue (BMAT) **(E)**, triglyceride (TG) **(F)**, total cholesterol (TC) **(G)**, high density lipoprotein-cholesterol (HDL-C) **(H)**, low density lipoprotein-cholesterol (LDL-C) **(I)**, glucose **(J)**, and insulin **(K)** in each PCOS subgroup were displayed with blue scale heat map, and the *p* values of the inter-subgroup comparisons were shown accordingly.

Values of the adiposity indices and biochemistry measurements were compared between the overweight and obese PCOS patient subgroups divided between those with normal values of androgens, LH, and LH/FSH ratio and those with abnormal values, respectively (**Figure 1**). None of the adiposity indices or biochemistry measurements were significantly different between the normal and abnormal TT subgroups. BMI (**Figure 1A**) and insulin (**Figure 1K**) were higher (*p* = 0.003—0.013) in the subgroups with abnormal FT and abnormal SHBG. Moreover, in the subgroup with abnormal SHBG, there was a higher VAT area (*p* = 0.003) (**Figure 1B**) and lower HDL-C (*p* < 0.001) (**Figure 1H**). Glucose (**Figure 1J**) was lower (*p* = 0.019) in the subgroup with abnormal LH, and insulin (**Figure 1K**) was lower (*p* = 0.011 and *p* = 0.049) in the subgroups with abnormal LH and abnormal LH/FSH ratio. There was no significant difference in BMAT between any of the subgroups (**Figure 1E**).

### The relationships between bone marrow adipose tissue and abdominal adiposity indices and biochemistry in overweight and obese PCOS patients

**Figure 2:**
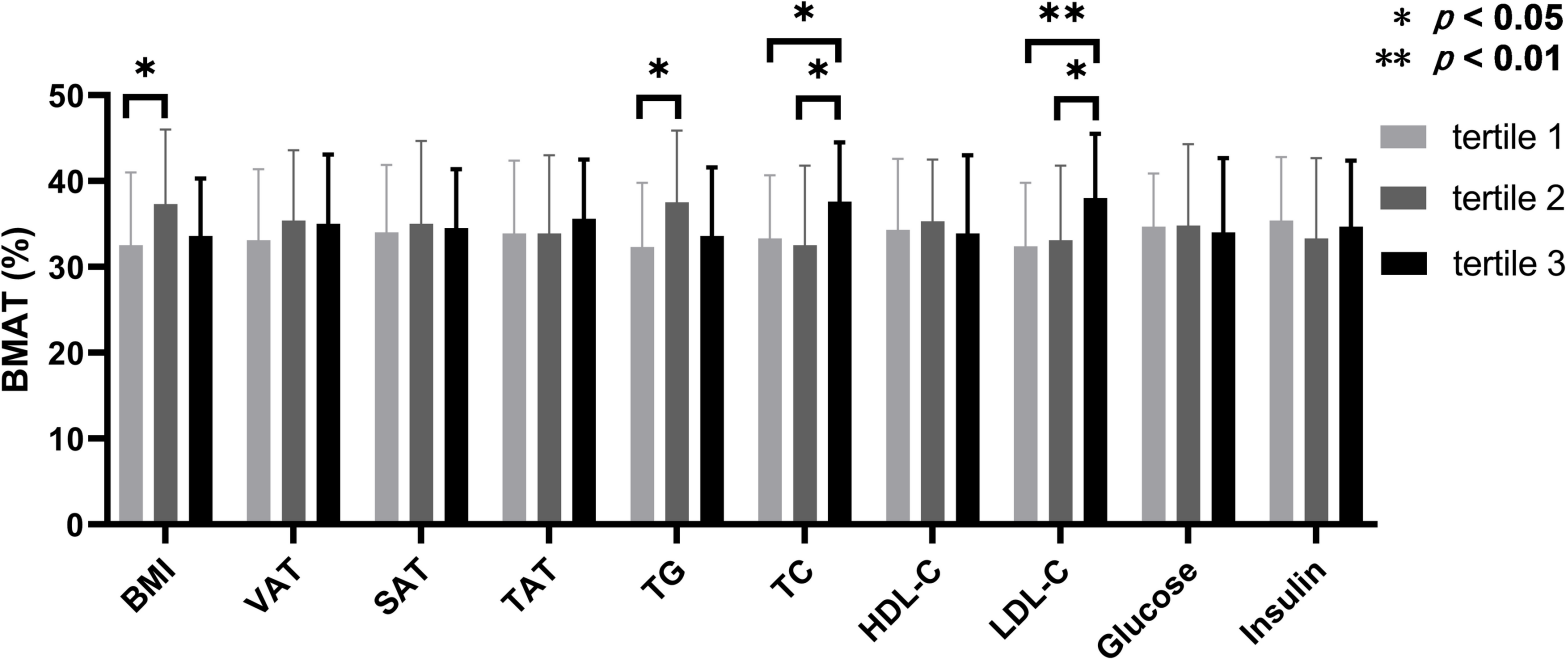
Comparisons of bone marrow adipose tissue between the tertiles of body adiposity indices and biochemistry measurements. The overweight and obese PCOS patients were stratified according to the tertiles of body mass index (BMI), visceral adipose tissue (VAT) area, abdominal subcutaneous adipose tissue (SAT) area, abdominal total adipose tissue (TAT) area, triglyceride (TG), total cholesterol (TC), high density lipoprotein-cholesterol (HDL-C), low density lipoprotein-cholesterol (LDL-C), glucose, and insulin, respectively. Bone marrow adipose tissue (BMAT) were compared between the tertiles of each index or parameter.

According to the inter-tertile comparisons, BMAT in the upper tertiles of TC and LDL-C were significantly higher compared to the lower and middle tertiles, and BMAT in the middle tertiles of BMI and TG were significantly higher than that in the lower tertiles (**Figure 2**). For all the other biochemistry measurements and all the abdominal adiposity indices, no significant difference of BMAT was detected between the tertiles (**Figure 2**).

**Table 3** shows the Pearson and partial correlations between BMAT and abdominal adiposity indices and biochemistry measurements in the overweight and obese PCOS patients. Only LDL-C showed a statistically significant correlation with BMAT (r = 0.253—0.263, *p* = 0.014—0.018) with or without adjustments for age and BMI (model 2), and additionally for FT, SHBG, and LH (model 3).

**Table 3:** Pearson and partial correlations between bone marrow adipose tissue and body adiposity indices and biochemistry measurements in overweight and obese PCOS patients.

	Pearson correlations		Partial correlations^a^		Partial correlations^b^		Partial correlations^c^	
	R	*p*	R	*p*	R	*p*	R	*p*
BMI	0.006	0.959	0.012	0.914	—	—	—	—
VAT area	0.075	0.489	0.087	0.427	0.106	0.336	0.088	0.431
SAT area	-0.037	0.736	-0.034	0.753	-0.059	0.589	-0.036	0.749
TAT area	-0.011	0.086	-0.006	0.953	-0.026	0.814	-0.007	0.950
TG	-0.068	0.529	-0.064	0.560	-0.066	0.549	-0.078	0.486
TC	0.20	0.063	0.210	0.052	0.210	0.054	0.209	0.060
HDL-C	-0.024	0.829	-0.030	0.787	-0.028	0.803	0.027	0.811
LDL-C	**0.253**	**0.018**	**0.263**	**0.014**	**0.263**	**0.015**	**0.262**	**0.017**
Glucose	0.053	0.629	0.066	0.544	0.066	0.550	0.057	0.613
Insulin	-0.013	0.908	-0.011	0.920	-0.018	0.870	-0.011	0.920

^a^Partial correlations adjusted for age;

^b^Partial correlations adjusted for age and BMI;

^c^Partial correlations adjusted for age, BMI, FT, SHBG, and LH.

BMI, body mass index; VAT, visceral adipose tissue; SAT, subcutaneous adipose tissue; TAT, total adipose tissue; TG, triglyceride; TC, total cholesterol; HDL-C, high density lipoprotein-cholesterol; LDL-C, low density lipoprotein-cholesterol.

The bold values mean statistically significant (p < 0.05).

### Contributions of sex hormones to elevated bone marrow adipose tissue in overweight and obese PCOS patients

Multivariate linear regression analysis showed that FSH was an independent risk factor (β = 0.284, *p* = 0.009) for the increase of BMAT in overweight and obese PCOS patients even after adjusting for age, BMI, and LDL-C (**Table 4**), while estradiol was an independent protecting factor (β = -0.226, *p* = 0.039). Neither androgens nor LH or LH/FSH ratio were associated with BMAT before or after adjusting for confounders (**Table 4**).

**Table 4:** Multivariate linear regression analysis showing the sex hormonal factors determining bone marrow adipose tissue in overweight and obese PCOS patients.

	Unadjusted		MV adjusted^a^		MV adjusted^b^	
	β	*p*	β	*p*	β	*p*
FSH	**0.311**	**0.003**	**0.313**	**0.004**	**0.284**	**0.009**
LH	0.098	0.369	0.098	0.372	0.131	0.234
LH/FSH ratio	-0.067	0.536	-0.068	0.534	-0.027	0.808
Estradiol	**-0.261**	**0.015**	**-0.260**	**0.016**	**-0.226**	**0.039**
Progesterone	-0.143	0.188	-0.141	0.197	-0.187	0.089
TT	0.138	0.203	0.135	0.217	0.124	0.262
FT	0.117	0.281	0.117	0.284	0.092	0.407
SHBG	-0.133	0.220	-0.136	0.215	-0.102	0.354
Prolactin	-0.055	0.613	-0.065	0.555	-0.078	0.480

^a^MV model adjusted for age and BMI;

^b^MV model adjusted for age, BMI, and LDL-C.

FSH, follicle stimulating hormone; LH, luteinizing hormone; LH/FSH, luteinizing hormone/follicle stimulating hormone; TT, total testosterone; FT, free testosterone; SHBG, sex hormone-binding globulin.

The bold values mean statistically significant (p < 0.05).

**Figure 3:**
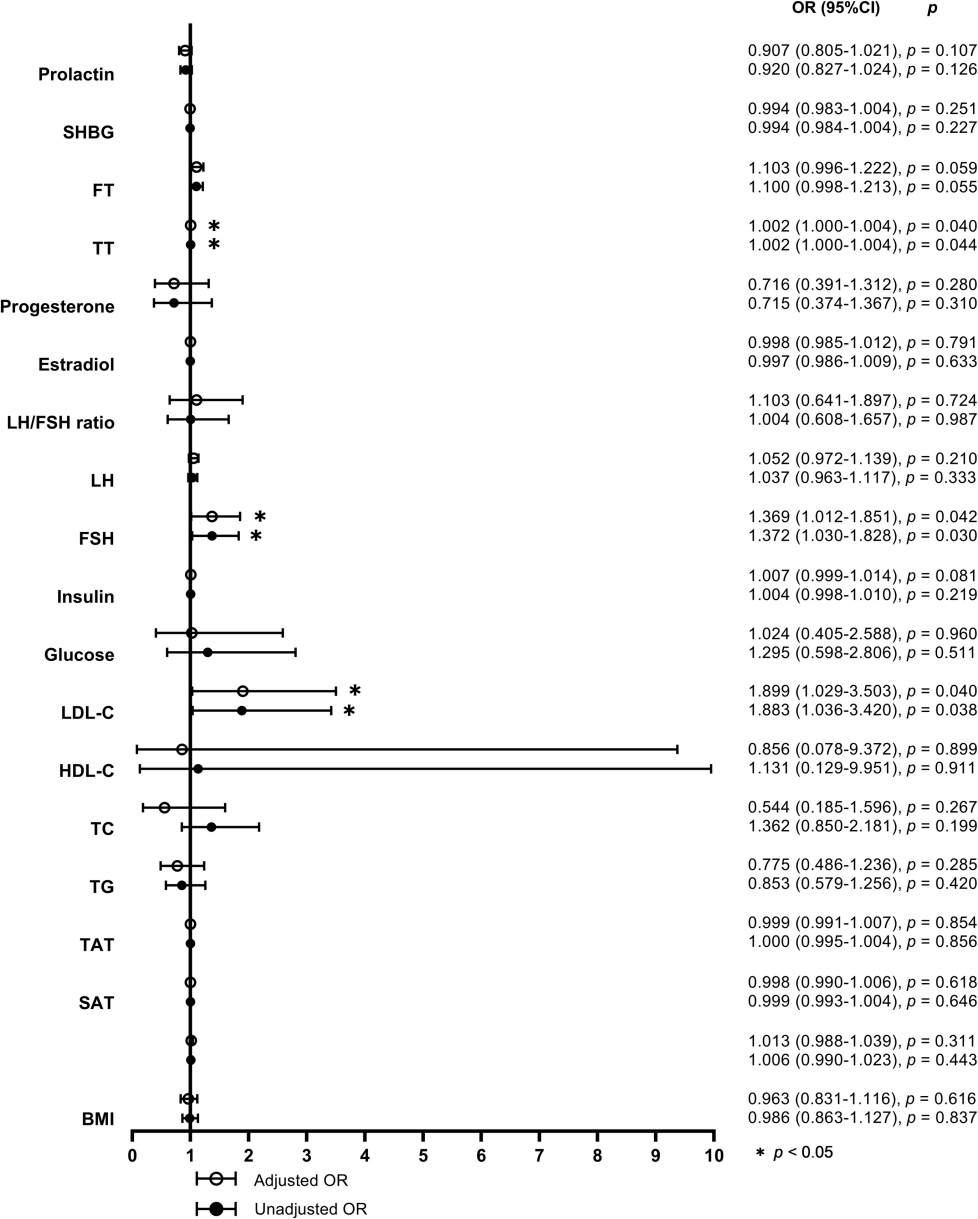
Forest plot of the unadjusted and adjusted odds ratios for potential determinants of elevated bone marrow adipose tissue. The odds ratios (ORs) for elevated bone marrow adipose tissue (BMAT) were calculated by univariate logistic regression models without (model 1) and with adjustments for age, body mass index (BMI), and low density lipoprotein-cholesterol (LDL-C) (model 2) for each unit increase of BMI (adjusted for age and LDL-C in model 2), visceral adipose tissue (VAT) area, abdominal subcutaneous adipose tissue (SAT) area, abdominal total adipose tissue (TAT) area, triglyceride (TG), total cholesterol (TC), high density lipoprotein-cholesterol (HDL-C), LDL-C (adjusted for age and BMI in model 2), glucose, insulin, follicle stimulating hormone (FSH), luteinizing hormone (LH), ratio of luteinizing hormone to follicle stimulating hormone (LH/FSH ratio), estradiol, progesterone, total testosterone (TT), free testosterone (FT), sex hormone-binding globulin (SHBG), and prolactin, respectively.

When defined by the 90% upper limit of the BMAT range of the controls, BMAT was considered elevated if BMAT was higher than 38%. A total of 27 (31%) of the overweight or obese PCOS patients were found with elevated BMAT. By unadjusted and adjusted univariate logistic regression analysis involving all the sex hormones, body adiposity indices, and biochemistry, LDL-C, FSH, and TT were found to be risk factors for the occurrence of elevated BMAT (**Figure 3**). For each unit increase in LDL-C, there was an 89.9% (*p* = 0.040) higher risk of an overweight or obese PCOS patient having elevated BMAT, after adjusting for age and BMI. After adjusting for age, BMI, and LDL-C, there was a 36.9% (*p* = 0.042) higher risk of an overweight or obese PCOS patient having elevated BMAT for a one-unit increase in FSH. There was only a small increase in risk (OR = 1.002, *p* = 0.040-0.044) for each unit increase of TT, before and after adjusting for cofounders. The cut off values of LDL-C, FSH, and TT for diagnosing elevated BMAT were 3.47 mmol/L, 4.925 IU/L, and 378.275 pg/mL, respectively. These three risk factors were transformed into dichotomous variables according to their cut off values and the results of multivariate binary logistic regression analysis are presented showing the predictors of elevated BMAT in overweight and obese PCOS patients (**Table 5**). FSH (OR = 12.043, *p* = 0.023) was the strongest predictor for elevated BMAT in overweight and obese PCOS patients, followed by TT (OR = 4.005, *p* = 0.019) and LDL-C (OR = 3.962, *p* = 0.013).

**Table 5:** Multivariate binary logistic regression analysis for predictors of elevated bone marrow adipose tissue in overweight and obese PCOS patients.

	Elevated BMAT (>38%)			Nagelkerke R^2^
	OR	95% CI	*p*	
LDL-C(≥3.47 mmol/L)	3.962	1.343—11.687	**0.013**	0.343
FSH(≥4.925 IU/L)	12.043	1.406—103.122	**0.023**
TT (≥378.275 pg/mL)	4.005	1.257—12.760	**0.019**

OR, odds ratio; CI, confidence interval; BMAT, bone marrow adipose tissue; LDL-C, low density lipoprotein-cholesterol; FSH, follicle stimulating hormone; TT, total testosterone. The bold values mean statistically significant (p < 0.05).

## Discussion

This study is the first to explore the phenotype of BMAT in overweight or obese young PCOS patients and the relationships between the increased BMAT and hyperandrogenism, obesity, and metabolic abnormalities in PCOS. In doing so, we found that BMAT of the L2 to L4 vertebra is increased by an average of 5.6% ( ± 11.3%) in overweight and obese PCOS patients compared to the age-matched controls. However, the increase in BMAT was unrelated to the majority of abnormal sex hormones in PCOS, including FT, SHBG, LH, and LH/FSH ratio, nor to the exacerbation of body adiposity or metabolic parameters related to those abnormal sex hormones. LDL-C was the only metabolic risk factor for an elevated BMAT and was positively correlated with BMAT. However, LDL-C levels were unrelated to the abnormal sex hormones in overweight and obese PCOS patients. FSH was independently associated with the increase in BMAT and was the strongest predictor for an elevated BMAT even after adjusting for age, BMI, and LDL-C, although its level remained normal or slightly decreased in overweight or obese PCOS patients. TT was also shown to be a risk factor for elevated BMAT but lacked a linear relationship with BMAT. An abnormally elevated TT was unrelated to the exacerbation of body adiposity or metabolic parameters.

The most likely etiology of PCOS is functional ovarian hyperandrogenism. Hyperandrogenism causes a series of pathophysiological changes in PCOS patients, including insulin resistance (21), hyperinsulinemia, and dyslipidemia (22), and conversely, hyperinsulinemia and obesity in PCOS play an important role in the establishment of hyperandrogenism (23). Hyperinsulinemia stimulates the production of ovarian androgens through its effects on the fully functional post-receptor MAP-K insulin pathway (24), and insulin also suppresses the hepatic production of SHBG, thereby accounting for the suppressed serum levels of SHBG in PCOS (25, 26). Other growth factors, inflammatory factors, and adipokines resulting from obesity may further stimulate excess ovarian androgen production or inhibit the aromatization of androgens to estrogens (27, 28). In our present study, BMI and insulin in overweight or obese PCOS patients were found to be higher in the subgroup with elevated FT or reduced SHBG.

Abdominal obesity is a condition related to hyperandrogenism with testosterone increased lipogenesis in visceral fat deposits in women (10, 29). According to a meta-analysis, increased abdominal subcutaneous and visceral fat accumulation was identified in women with PCOS compared to the BMI-matched healthy controls, but this difference disappeared when imaging methods were restricted to MRI or CT (30). In our present study, abdominal visceral and subcutaneous adipose tissue of overweight or obese PCOS patients were compared to their age-matched healthy controls, but there were no comparisons with BMI-matched controls. Considering the obvious difference in BMI between the two groups, it is unsurprising that the abdominal SAT quantified by SAT area was significantly higher in the PCOS patients. However, as unexpected, abdominal VAT quantified by VAT area was not significantly different between overweight or obese PCOS patients and age-matched controls. Despite the absence of increase in overweight or obese PCOS patients, abdominal VAT was found to be higher in the subgroup with reduced SHBG.

Androgen excess has a deleterious impact on metabolic homeostasis in women with PCOS, acting on different metabolic tissues such as the adipose tissue, liver, muscle, and the pancreas. A decrease in HDL-C and an increase in TG levels are well known lipid profile characteristics in women with PCOS (31, 32), and the free androgen index (FAI) positively correlates with TC, TG, and homeostasis model assessment for insulin resistance (HOMA-IR), and negatively with HDL-C (33, 34). PCOS women with elevated FT levels have an adverse metabolic phenotype (35). Other sex steroids, such as estradiol and TT, showed no significant relationship with metabolic parameters (BMI, waist circumference, glycemia, insulin, HOMA-IR, TC, TG, LDL-C, HDL-C) (33). The aforementioned findings are consistent with the results of this study, which showed lower HDL-C in the subgroup with reduced SHBG and higher insulin in the subgroup with elevated FT or reduced SHBG. Many studies have reported that LDL-C is increased in women with PCOS, but the reason for this is not clear yet. Obesity or overweight (and higher insulin resistance) is not the only reason for elevated LDL-C in PCOS. Increased LDL-C levels in women with PCOS may be related to hyperandrogenism or genetic factors (36–38). The exact relationship between hyperandrogenism and elevated LDL-C is still controversial due to heterogeneity among different studies. The meta-analysis conducted by Yang, et al. showed that there was a statistically significant difference in HDL-C between the PCOS/hyperandrogenism and PCOS/non-hyperandrogenism groups, but there was no significant difference in LDL-C between the PCOS/hyperandrogenism and PCOS/non-hyperandrogenism groups (39). We also did not find any significant difference in LDL-C between subgroups with different androgen levels in overweight or obese PCOS patients.

Although BMAT is poorly investigated in obese populations, it has been verified that young obese subjects have higher L4 marrow fat compared to normal-weight control subjects (40). BMAT in the long bones is also increased in the obese mouse model with spontaneous leptin disruption or a high-fat diet (41, 42). In response to metabolic variations, BMAT development differs from that of other fat depots in both humans and rodents. Serum LDL-C levels were found to be significantly correlated with vertebral BMAT, leading to an increase in BMAT in patients with type-1 diabetes and the middle-aged general population, and these results are consistent with ours in overweight and obese PCOS patients (43, 44). Statins are commonly used to lower cholesterol by competitively inhibiting 3-hydroxy-3-methylglutaryl-CoA (HMG-CoA) reductase, the rate-limiting enzyme of the mevalonate pathway, thus lowering LDL-C and raising HDL-C concentrations. Interestingly, statins have been reported to play protective roles in bone metabolism (45). Simvastatin also decreased Oil Red O staining and inhibited the gene expression of lipoprotein lipase (*LPL*) and peroxidase proliferator activated receptor gamma (*PPAR*γ) in a dose-dependent fashion. These results indicate that simvastatin has anabolic effects on bone that might be associated with the inhibition of adipocytic differentiation (46). Except for LDL-C, the associations between BMAT and other serum lipids reported in previous publications were not confirmed in our present study (40, 43, 44). The relationship between BMAT and metabolic abnormalities seems to be diverse and dependent on the disease characteristics, age, gender, and degree of obesity.

Regarding FSH, our findings confirmed the results in older postmenopausal women from the AGES- Reykjavik study, which showed that those with higher serum FSH had higher BMAT (47). In overweight and obese PCOS patients, FSH is the strongest predictor for an elevated BMAT, although its level was within the normal range. Lower endogenous estradiol was not significantly associated with higher BMAT in older postmenopausal women from the AGES- Reykjavik study (48). In previous clinical studies, exogenous estradiol has been reported to reduce BMAT in both premenopausal and postmenopausal women (49, 50). In the presents study, endogenous estradiol was negatively correlated with BMAT, but a decrease in estradiol did not induce an elevated BMAT in overweight or obese PCOS patients. Our findings imply that a fluctuation of estradiol within the normal range is not enough to induce elevated BMAT in premenopausal women. The effects of endogenous estradiol on BMAT seem to be variable among female populations at different estradiol levels or menstrual status.

To our knowledge, the AGES- Reykjavik study by Mistry et al. is the only one to investigate the relationships between androgens and BMAT in women, and none of the androgens, including FT, TT, and SHBG, showed a significant association with BMAT in older postmenopausal women (48). In the female rodent models of PCOS induced by testosterone propionate or dehydroepiandrosterone (DHEA), lower marrow fat volume was detected in the normal control group and the group with a shorter injection period, which demonstrated that the increase in BMAT was due to exogenous testosterone (14, 15). In overweight and obese PCOS patients, FT and SHBG were also not associated with increased BMAT, even though their levels increased in varying degrees, but TT was shown to be a risk factor for elevated BMAT despite the lack of a linear association with BMAT. Based on the above findings, we hypothesize that testosterone does not lead to the elevation of BMAT in women before its level reaches a certain threshold.

The main limitation of this study was that only 40 controls underwent CSE-MRI measurement for BMAT so that the threshold for an elevated BMAT was based on a relatively small reference sample size. In the future studies of BMAT, it will be necessary to establish a larger reference database involving different age groups and BMI levels. Second, due to the absence of a BMI-matched control group, we are unable to compare BMAT between PCOS and non-PCOS overweight or obese women. Third, we were unable to measure abdominal adipose tissue volume in this study, and therefore the identification of an evaluated abdominal fat mass using abdominal adipose tissue area may not be sufficiently accurate. In addition, Anti-Mullerian hormone (AMH) is a unique dimeric glycoprotein and also plays an important role in the pathophysiology of PCOS. Women with PCOS are noted to have higher levels of AMH and increased levels of serum AMH correlate highly with PCOS, polycystic ovarian morphology, hyperandrogenism, and oligo/amenorrhea (51, 52). Serum AMH has potentially important values in the diagnosis and evaluation of PCOS. In this group of women with PCOS, the relationships between AMH level and increased BMAT were not assessed because of the lack of AMH measurements. Despite these limitations, this study found an increase in BMAT in overweight and obese PCOS patients, as well as a link to hyperandrogenism, obesity, and metabolic abnormalities and its risk factors, and it paves the way for future research to clarify the mechanisms of the effects of androgens and cholesterol on BMAT.

## Conclusion

BMAT is increased in overweight or obese PCOS patients compared to the age-matched controls, and the increase in BMAT was unrelated to the majority of abnormal sex hormones in PCOS or the exacerbation in body adiposity or metabolic parameters related to hyperandrogenism. LDL-C and FSH were independently associated with the increase in BMAT. Although it lacked a linear relationship with BMAT, TT was also a risk factor for elevated BMAT besides LDL-C and FSH.

## Data availability statement

The raw data supporting the conclusions of this article will be made available by the authors, without undue reservation.

## Ethics statement

The studies involving human participants were reviewed and approved by Regional Ethics Committe of Beijing Obstetrics and Gynecology Hospital, Capital Medical University, and Regional Ethics Committe of Beijing Jishuitan Hospital. The patients/participants provided their written informed consent to participate in this study.

## Author contributions

The first authorship of LX and MM is of an equal rank. LX and MM designed the study and prepared the first draft of the paper. XL contributed the experimental work and data collection. GB edited the draft. KZ was responsible of the statistical analysis of the data. XC and XR supervised the study and paper organization. All authors agree to be accountable for the work and to ensure that any questions relating to the accuracy and integrity of the paper are investigated and properly resolved. All authors contributed to the article and approved the submitted version.
